# Biocatalytic synthesis of phenyl benzoate esters using the amide ligase ClxA

**DOI:** 10.1039/d5cb00205b

**Published:** 2025-10-08

**Authors:** Alexander Ascham, Qingyun Tang, Ian J. S. Fairlamb, Gideon Grogan

**Affiliations:** a Department of Chemistry, University of York Heslington York YO10 5DD UK gideon.grogan@york.ac.uk

## Abstract

The synthesis of ester bonds using lipases is one of the most frequently performed reactions in biocatalysis, yet examples of the enzymatic synthesis of phenyl benzoate esters are comparatively rare. In this report we show that the ligase ClxA, from *Clostridium cavendishii*, initially reported to have roles in amide bond formation in the biosynthesis of benzoxazole antibiotics, is an effective catalyst for the formation of phenyl benzoate esters from acid and phenol substrates using ATP in an aqueous medium. The structure of ClxA in a complex with both AMP and 3,4-aminohydroxybenzoic acid was determined by X-ray crystallography to 2.15 Å resolution and used as a platform to engineer the enzyme to create variants N226L and K140A possessing broader substrate specificity for ester formation, and also the ability to enable the synthesis of native amide product oligomers.

## Abstract 2

The ligase ClxA catalyses the synthesis of phenyl benzoate esters from benzoic acids and phenols and can be engineered for broadened substrate specificity.

## Graphical abstract

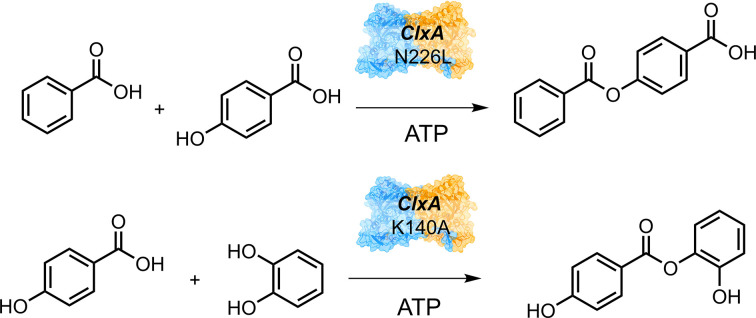

## Introduction

**Scheme 1:**
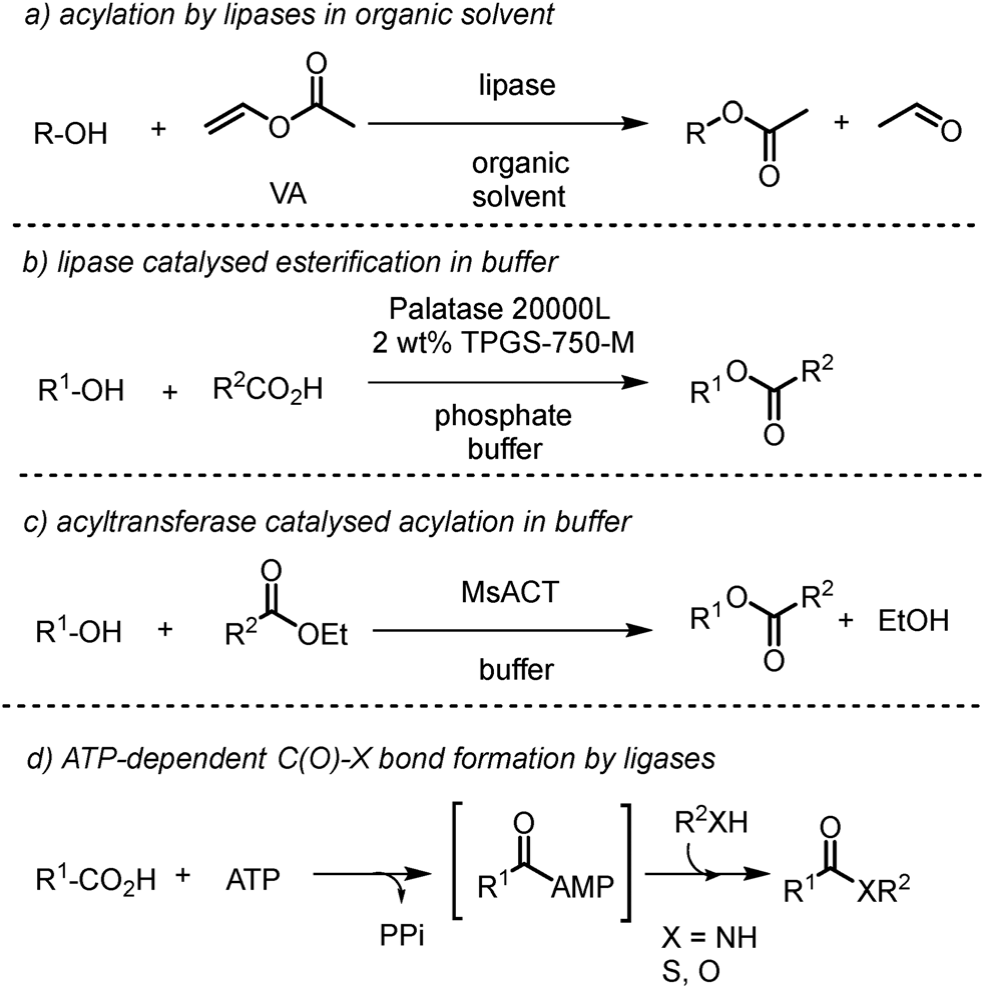
C(O)–X ((X = O, S or N)) bond formation using enzymes. (a) Lipase-catalyzed synthesis of esters in organic solvent using an acyl donor (vinyl acetate, VA); (b) palatase 20 000L-catalyzed synthesis of esters in aqueous buffer;^5^ (c) acyl transferase-catalysed synthesis of esters in aqueous buffer; (d): C(O)–X bond synthesis using adenylases.

The synthesis of esters is one of the most significant reactions in organic synthesis and is often accomplished through the use of hydrolase enzymes such as lipases.^1–4^ In these reactions, a lipase is used in a ‘reverse hydrolysis’ mode in which the enzyme first reacts with a simple ester substrate, an acyl donor such as vinyl acetate, to form a covalent acyl enzyme intermediate on a catalytic serine residue in the active site, followed by its breakdown by nucleophilic attack of an alcohol to form an ester (Scheme 1a). The use of an organic solvent prevents the hydrolysis of the ester product. Lipases can however, on occasion, catalyze ester formation effectively in water. In one recent example, the lipase Palatase 20 000L, which is derived from *Rhizomucor miehei*, catalyzed the synthesis of various esters from acid and alcohol precursors, when the enzyme is under micellar conditions, using the additive TPGS-750-M in phosphate buffer (Scheme 1b).^5^ In addition, the recent discovery of water active ‘acyl transferase’ enzymes, such as *Ms*ACT from *Mycobacterium smegmatis*,^6–9^ permits the synthesis of esters from acyl donors and alcohols in an aqueous medium (Scheme 1c). A common requirement for ester synthesis in synthetic chemistry and in Nature is the activation of the carboxylic acid partner. In many cases in Nature, when C(O)–X bond formation (X = N, O, S) is required for biosynthesis, the acid partner is activated, often through reaction with ATP, to form an adenylated intermediate (Scheme 1d), which is then attacked by a nucleophile, such as the deprotonated thiol of Coenzyme A, or the nitrogen of an amine, to form a thioester or amide respectively.^10^ While many ‘adenylases’ are only competent for the catalysis of adenylate formation, others, including the amide bond ligases^11^ recently studied by our group,^12–14^ and ester forming enzymes such as AcsD from *Pectobacterium chrysanthemi*,^15^ also actively catalyze the second step of C(O)–X (X = O, S or N) bond formation, and can therefore serve as selective and atom-economical biocatalysts for amide and oxo-ester bond formation. Despite the increasing recognition of their potential utility in amide bond synthesis, the application of adenylases in ester synthesis has not been widely reported, presumably because of the simplicity and effectiveness of existing lipase methods. However, there are instances where the availability of other enzymes for ester bond formation may prove useful, particularly where lipase substrate selectivity may be limiting, or where there is preference for coupling an acid and an alcohol directly in an aqueous synthetic medium. In this report we show that the ATP-dependent ligase ClxA from *Clostridium cavendishii*, which was previously reported to form amide bonds in the biosynthesis of benzoxazole natural products,^16^ is an effective biocatalyst for the ATP-dependent formation of phenyl benzoate esters from acid and alcohol precursors in an aqueous medium, and can also be engineered for altered substrate specificity and product outcomes, including the synthesis of native amide product oligomers.

## Results and discussion

### Substrate specificity of ClxA

**Scheme 2:**
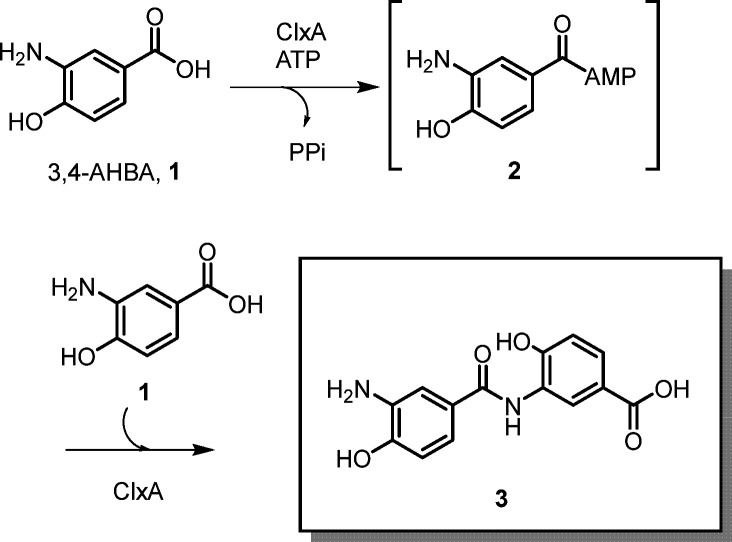
Reported reaction catalysed by ClxA from *Clostridium cavendishii*.

As part of ongoing studies into ligase enzymes that may have potential for preparative amide bond synthesis, our attention was drawn to recent work by the group of Hertweck,^16^ who reported the discovery of a pathway for benzoxazole synthesis in the anaerobic bacterium *Clostridium cavendishii*, leading to natural products named closoxazoles. A crucial part of this pathway was the ATP dependent coupling of two molecules of 3-amino-4-hydroxybenzoic acid (3,4-AHBA 1, Scheme 2) to form the amide product 3*via* adenylate intermediate 2. This step was reported to be catalyzed by an ATP-dependent ligase enzyme named ClxA.

ClxA was also reported to accept alternative substrates for coupling, including 3,5-dichlorobenzoic acid, which could be coupled to 3,4-AHBA 1 to form a precursor of an analog of the bioactive compound tafamidis.^16^ ClxA therefore presents as a possible biocatalytic tool for the synthesis of pharmaceutical-type amides, particularly those that use anilines as the amine partner, as these had proven to be poorer substrates for the amide bond synthetases McbA^12,13^ and ShABS^14^ studied by our groups previously.

**Scheme 3:**
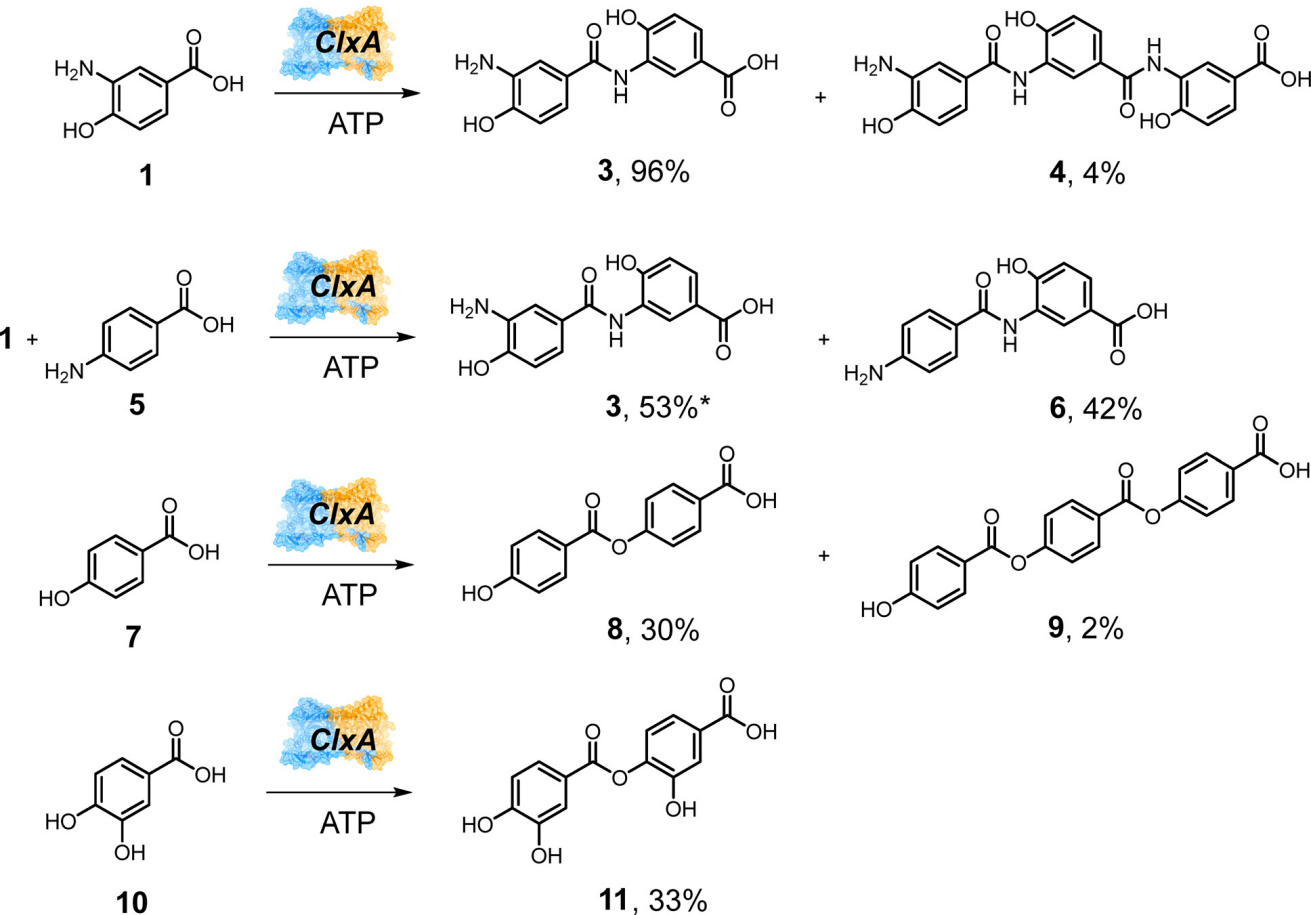
Formation of amide and ester products using ClxA% values refer to conversions as measured by HPLC. 200 μL scale reactions contained 50 mM KPi pH 7.5, with 10 mM acid, 10 mM alcohol/amine and 10 mM ATP. ClxA was added to a final concentration of 1 mg mL^−1^ and reactions were stopped after 24 h. *calculated from figures obtained from peak integration, although 3 and 5 were not fully resolved (Fig. S4A).

In order to assess the broader substrate specificity of ClxA, the gene was codon-optimised for expression in *E. coli* and expressed using the pET-YSBLIC-3C vector developed in our groups^17^ (SI Section S1). The protein was purified using nickel affinity (NiNTA) and size exclusion chromatography (SEC) (SI Section S2) and assayed against 3,4-AHBA 1 with ATP to confirm native activity using an HPLC assay (SI Sections S3–S5). ClxA was shown to catalyze smooth transformation of 1 to 3 (96% conversion), but also a trimeric product 4 (4%), a further activity of ClxA that was also noted by Hertweck and co-workers (Scheme 3 and Fig. S3).^16^ No products were observed with reactions conducted in the absence of either ClxA or ATP. A preliminary screen of alternative substrates was also performed on an analytical scale (Scheme 3), in which a number of constraints on the limitations of substrate specificity, with respect to both the activated acid ‘donor’ and the amine ‘acceptor’ in ClxA was revealed.

In addition to the dimer and trimer products 3 and 4 obtained with 1, *para*-aminobenzoic acid 5 was also accepted as a substrate with 1 in a reaction that gave native product 3 with 53% conversion (Fig. S4), but also the hetero-coupled dimer 6 (42%), confirming observations made by Hertweck and co-workers.^16^ Minor amounts of 4 and a trimer (not shown) formed from 6 and another molecule of 1 were also observed in the MS analysis. However, when ClxA was incubated with ATP and 5 alone, only the adenylate of 5 (*m*/*z* 466.97 [M + H]^+^) was detected. Likewise, incubation of 3,4-diaminobenzoic acid (not shown), with ATP and ClxA did not give rise to any coupled products. Intriguingly, incubation of ClxA with *para*-hydroxybenzoic acid 7 alone with ATP gave dimer and trimer products 8 and 9 with conversions of 30% and 2% respectively; the first indication that ClxA could form esters (Fig. S5). Interestingly, the ClxA homolog PfxC from *Pyxidicoccus fallax*, which also catalyses the ATP-dependent ligation of two molecules of 1 in the synthesis of closoxazoles, was also very recently reported to catalyse the ligation of two molecules of 7 to give the dimeric ester product 8.^18^ This critical observation with ClxA was confirmed through further incubation of the enzyme with 3,4-dihydroxybenzoic acid 10 alone with ATP, giving ester product 11 with 33% conversion (Fig. S6). The results with ClxA appeared to identify the 4-hydroxyl group of 1, 7 and 10 as an important determinant of acceptor recognition.

The synthesis of phenyl benzoates from their phenol and benzoic acid precursors in an aqueous medium presents an alternative opportunity for ester synthesis in biocatalysis, as rarely have such reactions been performed previously with lipases or acyl transferases. This is potentially useful as the phenyl benzoate motif occurs in a number of widely used pharmaceuticals, including the analgesics phenylsalicylate and benorilate and the serine protease inhibitor nafamostat.

**Scheme 4:**
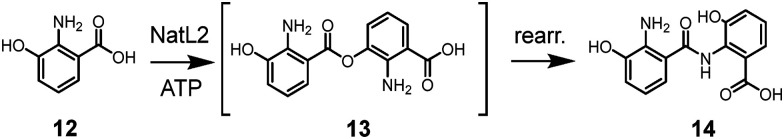
ATP-dependent coupling of 3-hydroxyanthranilic acid 3-HAA 12 by NatL2 to give amide 14*via* ester intermediate 13.^18^

The generation of esters by ClxA prompts further consideration of its substrate specificity and catalytic action. The structure of the ClxA product formed from homo-coupling of 1 as an amide was determined unambiguously by Hertweck and co-workers using NMR, HPLC retention times and MS/MS spectra against chemically synthesised standard compounds.^16^ Our analysis is in agreement with these findings (SI Fig. S3). However, a previous study on a related ligase NatL2 demonstrates that, in a homocoupling reaction between two molecules of 2-amino-3-hydroxybenzoic acid (3-hydroxyanthranilic acid 3-HAA 12, Scheme 4), it is in fact the ester 13 that is formed as a first product, followed by a rearrangement that occurs *via* a tetrahedral hemiorthoamide to form the amide product 14.^19^ As the aromatic amine and hydroxyl in 3,4-AHBA 1 are also arranged in an *ortho*-substitution pattern, as with 3-HAA 12, then rearrangement of an initial ester to an amide also seems feasible, notwithstanding the proposed role of the carboxylate *ortho*- to the amine in 12 as a base in the proposed NatL2 mechanism of ester-amide rearrangement.

### Structure of ClxA

The observations on both chemoselectivity and substrate specificity of ClxA prompted us to initiate structural studies as a precursor to structure-guided mutagenesis. Two datasets for ClxA were obtained (SI Section S6): The first was an *apo*- structure with no ligands bound; the second was obtained following co-crystallisation with both ATP and 3,4-AHBA 1 and features the ligand density described below. In each of the two datasets there were four molecules in the asymmetric unit. The monomeric structure from the *apo* dataset was analysed using the DALI server^20^ and showed greatest similarity, as anticipated, to NatL2 (6SIX;^19^ 29% sequence id (Fig. S8); Z-score of 41.5; rmsd of 2.6 Å over 437 Cα-atoms). The next most similar structures were those of phenylacetate coenzyme A ligases from *Bacteroides thetaiotaomicron* (4RVN; 26% sequence id; Z-score of 39.1; rmsd of 2.3 Å over 424 Cα-atoms) and *Burkholderia cenocepacia* J2315 (2Y4N;^21^ 19%; 37.6; 2.5 Å over 423 Cα-atoms); the latter presented in complex with a phenacetyl adenylate intermediate.

**Fig. 1:**
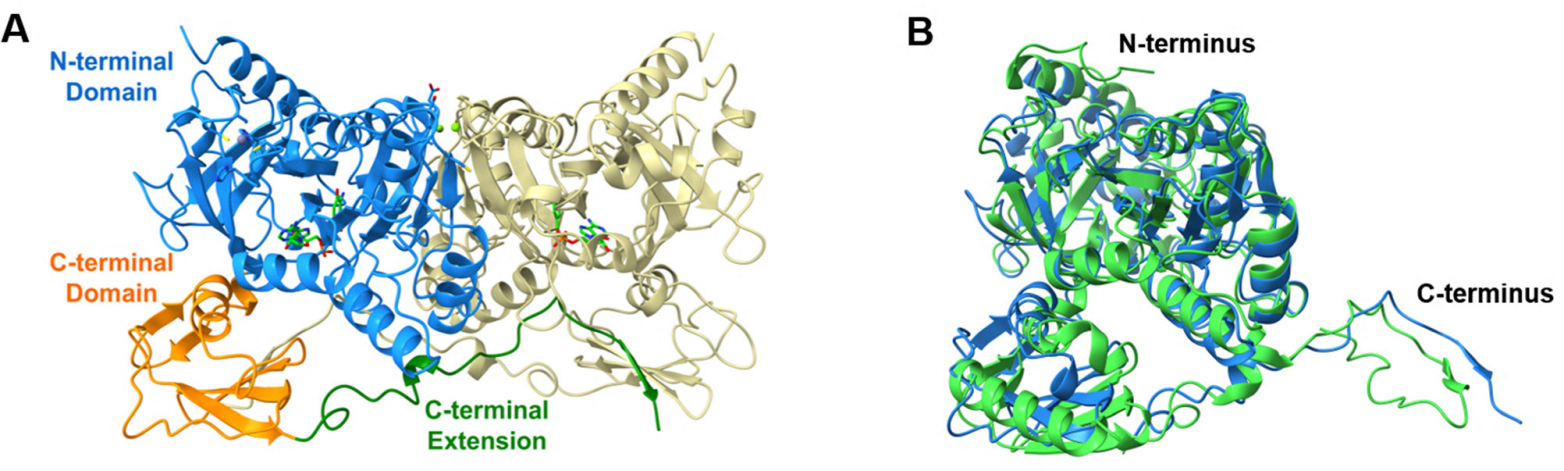
(A) Structure of ClxA dimer, highlighting domains and reciprocal domain sharing; (B) superimposition of ClxA monomer (blue) with NatL2 monomer (PDB code 6SIY, green).

The structure of NatL2 and ClxA are characteristic of a subset of adenylases in which the active sites are completed by reciprocal sharing of an extension to the C-terminal cap domain (Fig. 1A). The structural similarity of NatL2 to acyl-CoA ligases was described in detail previously,^19^ but this extension to the cap domain appears to be a feature restricted to ClxA, NatL2 and also the adenylase AjiA1 (6WUQ),^22^ which displays 90% amino acid sequence identity with NatL2. ClxA, with only 29% sequence identity to NatL2 (Fig. S8), features many local differences in secondary structure compared to NatL2: for example, NatL2 features a longer N-terminus and C-terminus (Fig. 1B) and an ordered helix (P373–R388) that is replaced by a shorter region of less well-defined helical character (D353 to N362) in ClxA.

**Fig. 2:**
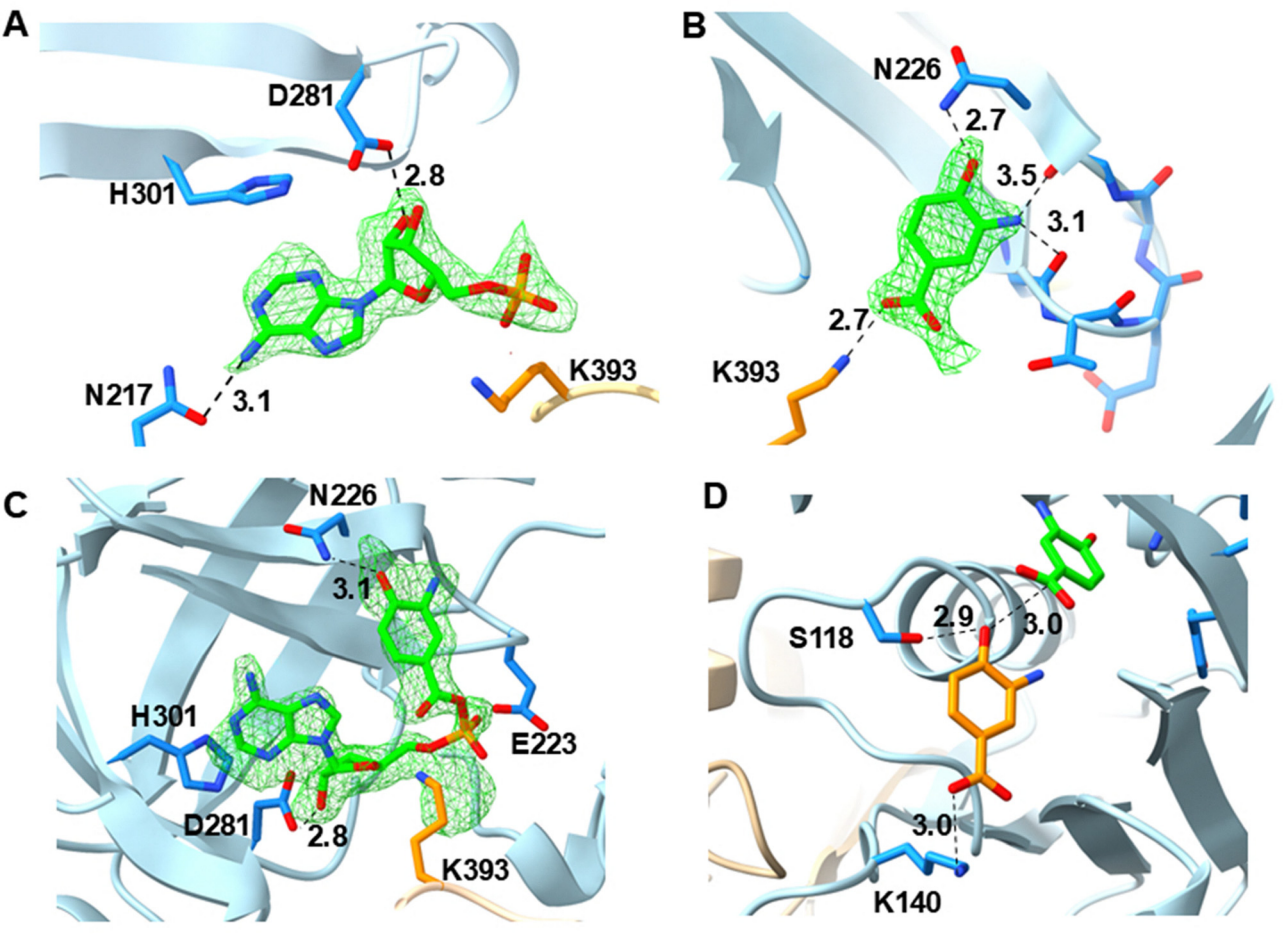
(A) Electron density for AMP in the ClxA active site; (B) binding of the acyl donor, 3-amino-4-hydroxybenzoic acid (3,4-AHBA 1) in the active site; (C) binding of the acyl adenylate of 1 in the active site; (D) AutoDock Vina generated binding conformation of 3 4-AHBA 1 in the ClxA acceptor site. Electron density in A, B and C corresponds to the *F*_O_*–F*_C_ omit map at a level of 3*σ* obtained prior to inclusion of the ligand in refinement. Selected interactions are shown as black dashed lines with distances in Ångstroms (Å).

The second dataset for ClxA featured omit density in each active site that was different in the monomers constituting each dimer (A/B and C/D). This dataset had been obtained by co-crystallising the protein with ATP and 3,4-AHBA 1. In subunits A and C, the density was best modelled as AMP plus 3,4-AHBA (Fig. 2A and B); in subunits B and D there was clear connective density between the AMP and 1, permitting modelling and refinement as the AMP-1 adenylate 2 (Scheme 1 and Fig. 2C). The determinants of nucleotide binding were mostly conserved between ClxA and NatL2: ClxA M198 (NatL2 P214), Y219 (Y236) and H301 (V319) form a pocket for the adenine base; D281 (D299) interacts with the ribose O2′ hydroxyl; phosphate recognition is performed by T222 (T239) and crucially K393 (K418) from the partner monomer, protruding into the active site from the C-terminal extension (Fig. 2A). However, the determinants of 3,4-AHBA 1 binding were different to those found in NatL2 for binding 3-HAA 12 (Fig. 2B). E235 (NatL2), which contacts the *meta*-hydroxyl of 12 was not present in ClxA, being replaced by Y218, which projects away from the active site; The *para*-hydroxyl group of 1 makes contact with the side chain of N226 in ClxA, a residue that is replaced by T243 in NatL2, and which was not directly involved in ligand binding. Interestingly, the NatL2 structure (6SIY) featured two molecules of 12 in the active site; the first primed for bonding to AMP, the second for reaction with the proposed adenylate intermediate. None of the residues making close contact to the acceptor ligand in 6SIY was conserved in ClxA, however, including T135 (ClxA P120), which in 6SIY makes an H-bond with the *ortho*-amino group of 12.

### Mutation of N226 and K140 in ClxA expands ester synthesis spectrum

The structure of ClxA in complex with both substrate 1 and adenylate intermediate 2 prompted an investigation of mutations designed to expand its substrate specificity. It was reasoned that mutation of asparagine N226, which was shown to make hydrogen bonding contact with the *para*-hydroxyl group of 1 (Fig. 2B), may be a significant determinant of acyl donor specificity, providing a hydrogen bond donor/acceptor to the *para*-position of the donor substrate. Accordingly, mutant plasmids carrying N226L and N226A mutants were prepared, and variants expressed and purified as for the wild-type enzyme (SI Section S11). These were then assayed against an expanded range of acyl donors using ATP.

**Scheme 5:**
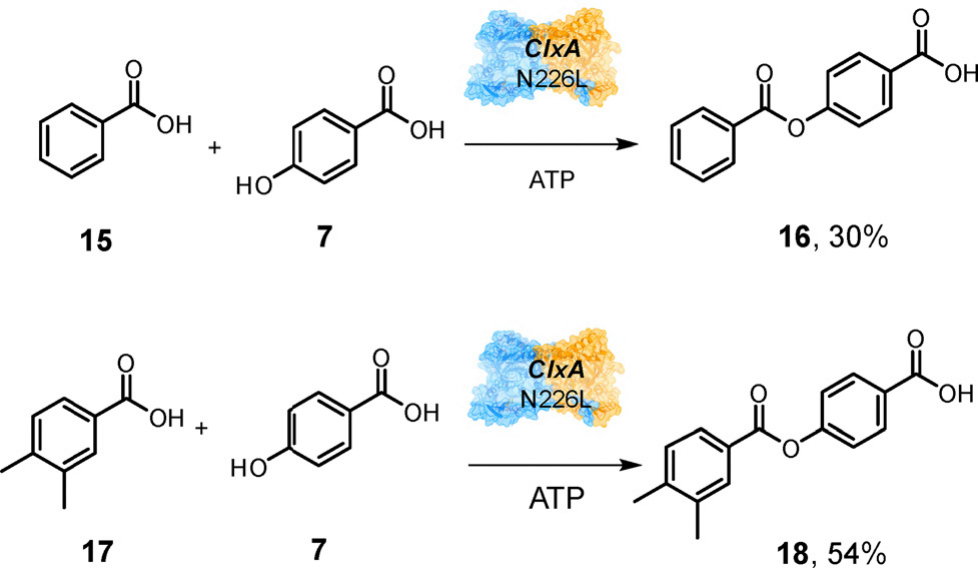
Substrate specificity of ClxA Mutant N226L. 200 μL scale reactions contained 50 mM KPi pH 7.5, with 10 mM acid, 10 mM alcohol/amine and 10 mM ATP. ClxA was added to a final concentration of 1 mg mL^−1^ and reactions were stopped after 24 h.

**Scheme 6:**
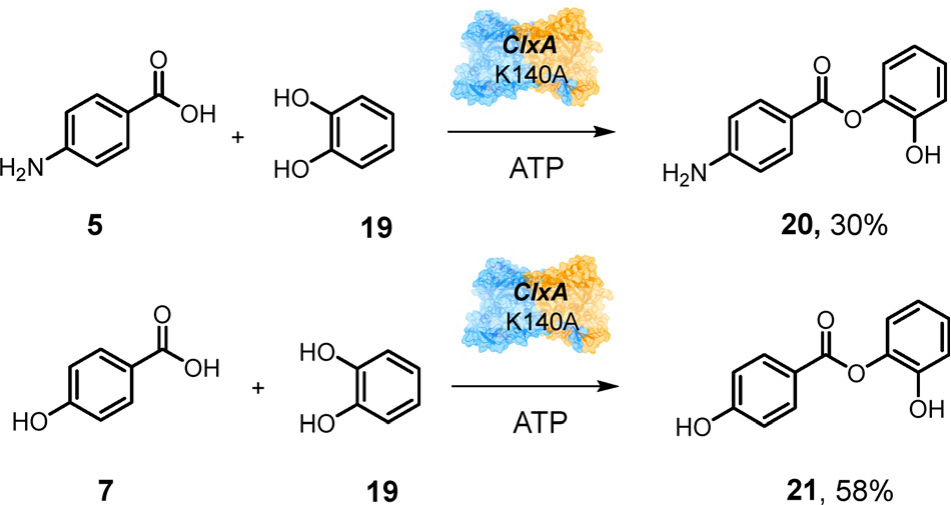
Substrate specificity of ClxA Mutant K140A. 200 μL scale reactions contained 50 mM KPi pH 7.5, with 10 mM acid, 10 mM alcohol/amine and 10 mM ATP. ClxA was added to a final concentration of 1 mg mL^−1^ and reactions were stopped after 24 h.

Although WT ClxA and N226A transformed benzoic acid 15 and 3,4-dimethylbenzoic acid 17 to complex mixtures of products, N226L catalysed hetero-coupling reactions with these substrates as acyl donors and *para*-hydroxybenzoic acid 7 as the acceptor to give products 16 (Fig. S10) and 18 (Fig. S11) with 30% and 54% conversions respectively (Scheme 5). Although we did not observe ligands in the acceptor site as observed in the NatL2 study,^19^ we modelled the putative acceptor ligand 1 into the equivalent site in ClxA using Autodock Vina^23^ and observed suggested interactions of the *para*-hydroxyl group with S118 and of the carboxylate with K140. Removal of the requirement for this ionic interaction through mutation of K140 to alanine gave a mutant K140A which now displayed activity for the coupling of both 5 and 7 with catechol 19, to give hetero-coupled products 20 (Fig. S12) and 21 (Fig. S13) with 30% and 58% conversion respectively (Scheme 6), where the wild-type exhibited only poor activity.

### ClxA K140A also enables the synthesis of oligoamides

**Scheme 7:**
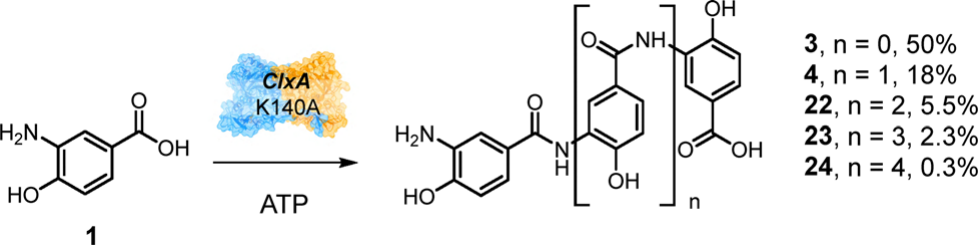
Oligoamide synthesis by ClxA K140 mutant% values indicate proportion of product in mixture as determined by HPLC.

In addition to the relaxation of acceptor specificity observed above, ClxA K140A also exhibited interesting behaviour when challenged with the native substrate of ClxA 3,4-AHBA 1 alone with ATP. In this instance, ClxA K140A catalyzed not only the formation of the native amide dimer 3 and trimer 4 products observed for the wild-type enzyme (Scheme 3), but minor, but significant, amounts of polyamides of 1 with *n* of up to 4 were also observed (Scheme 7 and Fig. S14). It is possible that these longer oligomers may have resulted from ‘off-enzyme’ reactions of relevant adenylates, released from the active site, with acceptor molecules in solution, as has been observed, for example with mutants of biotin protein ligases BirA^24^ and AaBPL.^25^

However, if the formation of longer oligomers is catalysed by the mutant enzyme, the change in polymer distribution might be partly attributed to the role of K140 in the recognition of the acceptor, as the smaller alanine residue may permit larger molecules to be accommodated in the acceptor access channel of the enzyme. In addition, in the wild-type, K140 may favour the binding of 1 as an acceptor through the ionic interaction with the carboxylate identified by modelling (Fig. 2D). When K140 is mutated to alanine, oligoamides may be more easily accommodated as acyl acceptors.

## Conclusions

The selective synthesis of C(O)–X (X = O, S or N) bonds in aqueous medium is an ongoing challenge to biocatalysis, especially where the substrate specificities of known enzymes is limiting. In this report we have shown that amide-bond forming enzymes can be engineered for the construction of bulky phenyl benzoate ester compounds from deactivated acid and alcohol partners to give molecules of pharmaceutical relevance, and using environmentally benign reagents and reaction conditions. This knowledge and structural insight concerning the catalytic promiscuity of C(O)–X bond ligases, serves as a platform for future engineering experiments to alter and improve upon these activities.

## Author contributions

IJSF and GG designed experiments and AA and QT performed experiments. The manuscript was written with contributions from all authors.

## Conflicts of interest

There are no conflicts to declare.

## Supplementary Material

CB-006-D5CB00205B-s001

## Data Availability

The data supporting this article have been included as part of the supplementary information (SI). Supplementary information: details of gene cloning and expression, protein purification, mutation, HPLC/MS analysis and X-ray crystallography data. See DOI: https://doi.org/10.1039/d5cb00205b.
