# Supplementary material for: Biocatalytic synthesis of phenyl benzoate esters using the amide ligase ClxA
Source: RSC Chem Biol. 2025 Oct 8;6(12):1879–84. doi: 10.1039/d5cb00205b (PMC12519233; doi:10.1039/d5cb00205b)
Supplement: CB-006-D5CB00205B-s001 [file CB-006-D5CB00205B-s001.pdf]

Supplementary Information (SI) for RSC Chemical Biology.
This journal is © The Royal Society of Chemistry 2025

Biocatalytic Synthesis of Phenyl Benzoate Esters Using the Amide Ligase ClxA

Alexander Ascham,[a] Qingyun Tang,[a] Ian J.S. Fairlamb[a] and Gideon Grogan[a]*

[a]Department of Chemistry, University of York, Heslington, York YO10 5DD U.K.

*gideon.grogan@york.ac.uk

Supporting Information

Table of Contents

1.	Cloning and Expression of ClxA	3
2.	Purification of ClxA	4
3.	Analytical–Scale Biotransformations	5
4.	HPLC Analysis	5
5.	HPLC and MS Analysis of WT ClxA-catalysed Biotransformations	6
6.	X-ray Crystallography	11
7.	Ligplot Diagram for Binding Site of the 3,4-AHBA adenylate	13
8.	Sequence Alignments	14
9.	AutoDock	14
10.	Ligplot Diagram for Modelled Binding Site for 3,4-AHBA 1 (Unl502) as an acceptor molecule	
11.	Site-Directed Mutagenesis	15
12.	HPLC/MS data for biotransformations by ClxA N226L and K140	16
13.	References	22

1. Cloning and Expression of ClxA

The ClxA gene encoding the amino acid sequence in **Figure S1** was codon optimised for *E. coli,* synthesised and ordered in a pET28a expression vector between *NdeI* and *HindIII* restriction sites, by Genscript.

MDSNSQLITKLNSALQIATKANFYKDRLGNIEIKSLDDFSKLPLTTKEDLRKLKPMEALTVDIE
DLFQYHESFGTTGEPVSTWLTEKDFNAYGDQLNEFGVNFKSTDIVLNRFPYAISVPAHIFTN
AIHKKGACVIPVSKASAISPLKRVANLIYKLRPSILTGIPDELIKLNKVAKFMDISLKDLGCIRAIC
TAGEMLSEGRKAKLESIFGAKVYNYYGCTECGNMAASCDEGHLHISKDFYVEILDPVTLKPV
KEGKGKIIVTTLNKEAFPMIRYDLGDIGEIKYEKCSCGNDRPVLIHHGREIDLIKTSKGTITFKE
LQEEIFKLPNSVVGDVFRVKIQNDEVIVECEADEELDNSNSNLNLPIEVKIKRFNHGEILNIDN
LIEIKPIAKPKYVEYVD

Figure S1. Amino acid sequence of ClxA used in this study.

The ClxA gene was subcloned into the pETYSBLIC3C vector using protocols previously described.[1] Briefly, the pETYSBLIC3C plasmid was linearised using PCR with Phusion HotStart II Polymerase (Thermo Fisher) according to the manufacturer's instructions. The ClxA gene was amplified using PCR, with 15 bp overhangs complementary to the pETYSBLIC3C plasmid ends (**Table S1**). The products were then digested using DpnI (New England Biolabs) according to the manufacturer's instructions. The amplicon and the linearised plasmid were purified (QIAquick PCR & Gel Cleanup Kit, Qiagen) and the ClxA gene was inserted into linearised pETYSBLIC3C using In-Fusion Snap-Assembly Master Mix (Takara Bio) according to the manufacturer's protocol. Stellar competent cells (Takara Bio) were transformed with the resulting plasmid and grown on LB-Agar plates supplemented with 35 µg mL^{-1} kanamycin. Plasmids were extracted and purified using a Wizard Plus Plasmid Purification kit (Promega) according to the manufacturer's protocol. Correct insertion was confirmed by Sanger Sequencing using standard T7 and T7-term primers (Eurofins).

**Primer**	**Primer Sequence 5' -> 3 '**
clxA Insert F	TTC CAG GGA CCA GCA ATG GAT AGT AAT TCA CAA CTA ATA ACA AAA C
clxA Insert R	GGA GAA GGC GCG CCT TTA ATC TAC GTA TTC CAC GTA TTT CG
LIC3C Lin F	CAA AGC CCG AAA GGA
LIC3C Lin R	TGC TGG TCC CTG GAA

Table S1. PCR primers used for subcloning ClxA gene into pETYSBLIC3C vector.

2. Purification of ClxA

E. coli BL21(DE3) competent cells (New England Biolabs) were transformed with pETYSBLIC3C-clxA and grown on LB-Agar plates supplemented with 35 µg mL^{-1} kanamycin at 37 °C overnight. A single colony was used to inoculate 10-50 mL LB broth supplemented with 35 µg mL^{-1} kanamycin and this culture incubated at 37 °C with 180 rpm shaking overnight. The overnight culture was then used to inoculate 1 L LB broth supplemented with 35 µg mL^{-1} kanamycin. The cell culture was grown at 37 °C with 180 rpm shaking until the OD_{600} reached 0.6 - 0.8, at which point expression was induced by the addition of isopropyl β-D-1-thiogalactopyranoside (IPTG) to a final concentration of 1 mM. The cells were then incubated at 16 °C with 180 rpm shaking for 18-22 h.

Cells were collected by centrifugation (5000 rpm, 30 min), resuspended in Buffer A (50 mM HEPES pH 7.5, 300 mM NaCl, 20 mM imidazole, 10 mM $MgCl_2$, 1% glycerol) and lysed using a high-pressure cell disruptor set to 27 kPa. Lysate was clarified by centrifugation (15000 rpm, 1 h), loaded onto a pre-equilibrated HisTrap FF Crude column (Cytiva) and washed with Buffer A. Protein was eluted by increasing the ratio of Buffer B (50 mM HEPES pH 7.5, 300 mM NaCl, 300 mM imidazole, 10 mM $MgCl_2$, 1% glycerol) from 0 to 100 % over 10 column volumes (CV). Fractions were analysed by SDS-PAGE and those containing ClxA were pooled and concentrated using a centrifugal protein concentrator (VivaSpin 20, 30 kDa MWCO).

ClxA was then further purified by size-exclusion chromatography (SEC) using a HiLoad 16/600 Superdex S75 pre-equilibrated with SEC buffer (50 mM HEPES pH 7.5, 300 mM NaCl, 10 mM $MgCl_2$, 1% glycerol). Fractions containing ClxA were pooled and concentrated. The protein was aliquoted, flash-frozen in liquid nitrogen and stored at -72 °C until use.

For crystallography, the His-tag was cleaved from protein following the Ni purification step. The protein was dialysed against 50 mM HEPES, 300 mM NaCl, 10 mM $MgCl_2$, 1 % glycerol and 1 mM DTT. The His-tag was cleaved using HRV3C protease (expressed in house) overnight at 4 °C. The cleaved protein was isolated using a HisTrap FF Crude column (Cytiva) pre-equilibrated with Buffer A and collecting the flow-through and a 5 CV wash with Buffer A. The cleaved protein was then purified by SEC as described above.

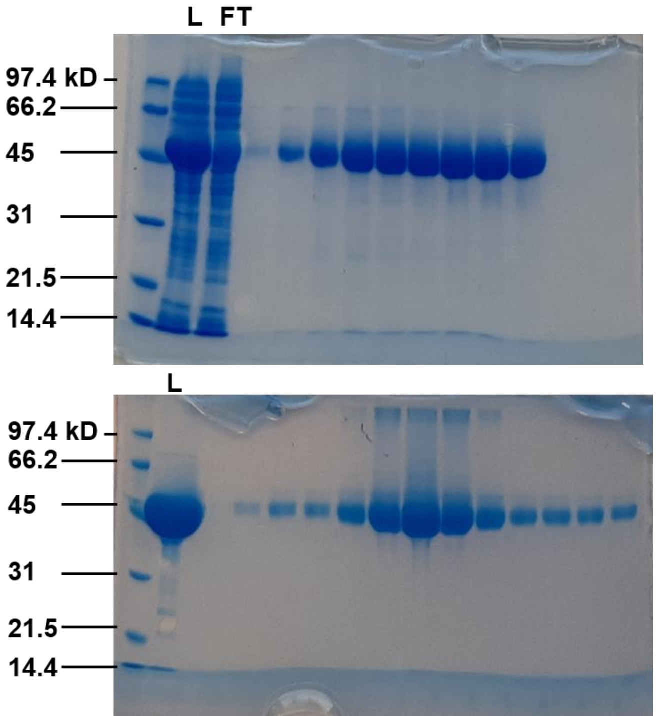

Figure S2. SDS-PAGE analysis of chromatography fractions during ClxA purification. **Top**: Nickel Affinity; **Bottom**: Size Exclusion Chromatography. **L** = loaded protein; **FT** = Flow-Through.

3. Analytical–Scale Biotransformations

100 mM stock solutions of substrates in DMSO were prepared. 100 mM stock solutions of ATP were prepared in water. 200 µL-scale reactions were constituted in 50 mM KPi pH 7.5, with 10 mM acid, 10 mM alcohol / amine and 10 mM ATP in a 1.5 mL Eppendorf tube. ClxA was added to a final concentration of 1 mg mL^{-1} and the reactions were incubated at 37 °C for 24 h with 750 rpm shaking. Reactions were quenched by the addition of 200 µL acetonitrile, centrifuged at 13000 r.p.m. to remove protein and analysed by HPLC / LC-MS.

4. HPLC Analysis

HPLC analysis was performed on an Agilent 1260 Infinity II HPLC fitted with a 1260 MCT UV detector and using an Agilent Poroshell EC-C18 3.0 x 150 mm 2.7-Micron column fitted with a 5 mm guard column, using the following eluents and gradients (**Table S2**).

Eluents: A: H_2O (+ 0.1 % (v/v) formic acid); **B**: Acetonitrile (+ 0.1 % (v/v) formic acid).

Flow rate: 400 µL min^{-1}

Gradient:

Time / min	% A	% B
0	90	10
15	10	90
17	10	90
18	90	10
22	90	10

Table S2. Gradient used for HPLC Analysis.

5. HPLC and MS Analysis of WT ClxA-catalysed Biotransformations

Reactions were performed on a 200 μL scale and contained 50 mM KPi pH 7.5, with 10 mM acid donor, 10 mM carboxylic acid donor, 10 mM amine acceptor and 10 mM ATP. ClxA was added to a final concentration of 1 mg mL^{-1} and reactions were stopped after 24 h. All chromatograms represent analysis of reactions after 24 h incubation. **A** – ATP/AMP.

A

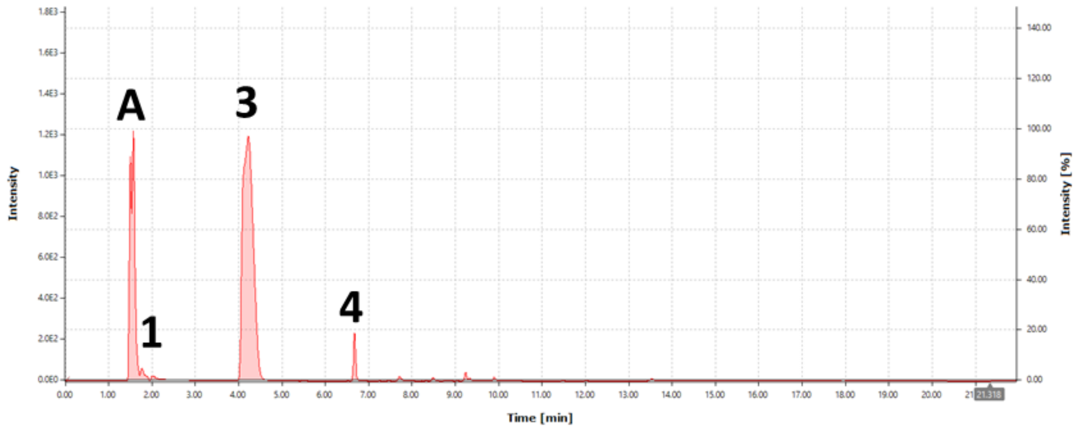

B

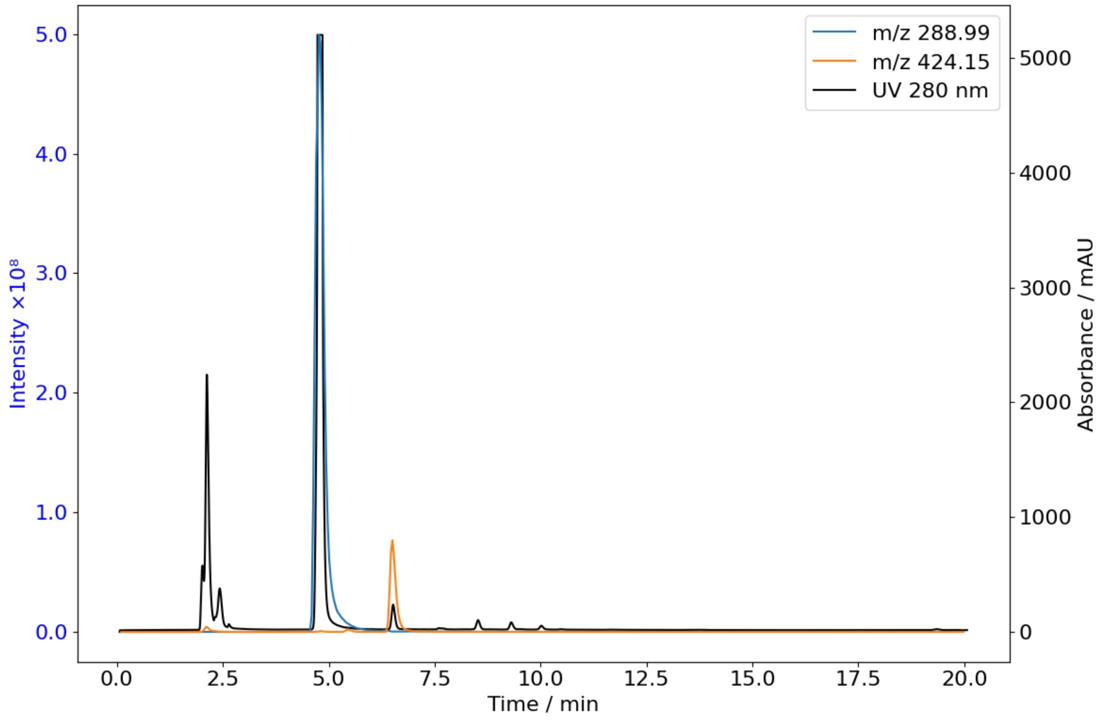

Figure S3: A. HPLC trace for ClxA-catalyzed homo-coupling of 3-amino-4-hydroxybenzoic acid **1** (**A** = ATP/AMP); **B**: Extracted Ion Chromatograms of products **3** *m/z* 288.99 $[M+H]^+$ (expected 289.07) and **4** *m/z* 424.15 $[M+H]^+$ (expected 424.11).

A

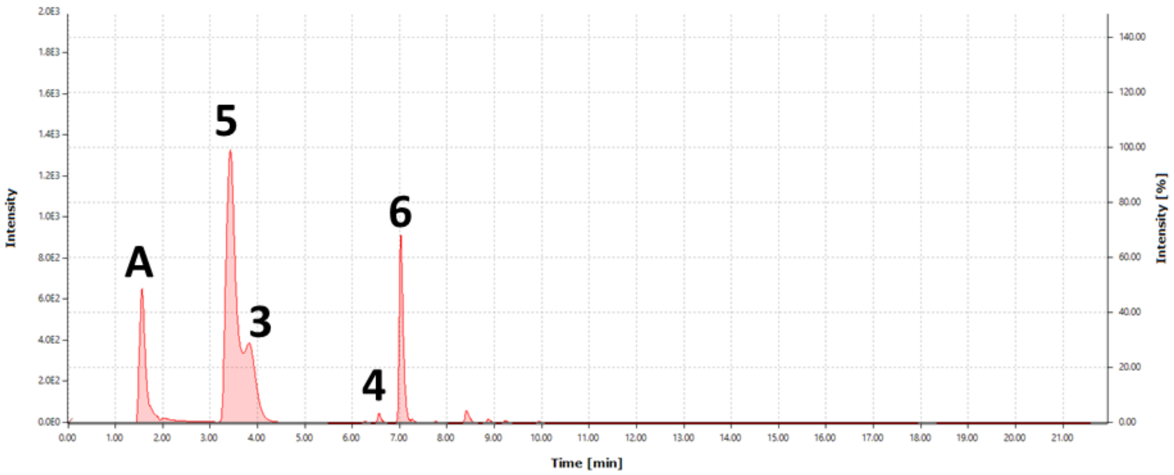

B

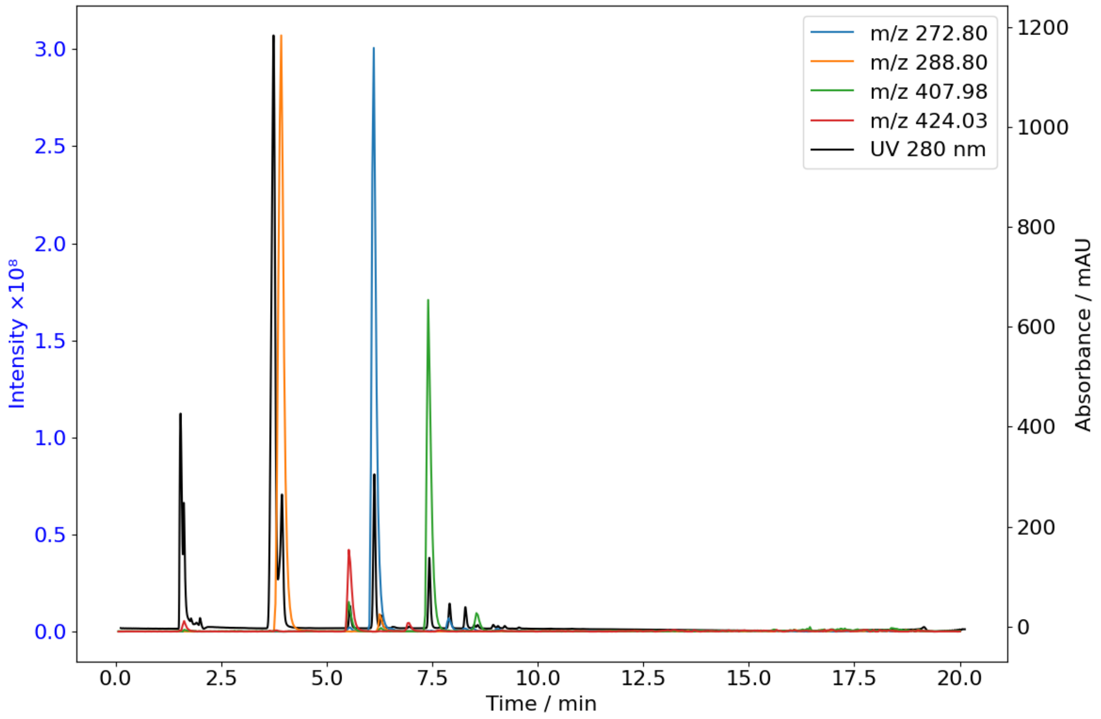

Figure S4. **A**: HPLC trace for ClxA-catalyzed reaction of 3-amino-4-hydroxybenzoic acid **1** and *para*-aminobenzoic acid **5** (**A** = ATP/AMP); **B** Extracted Ion Chromatograms of products **3** *m/z* 288.80 $[M+H]^+$ (expected 289.07), **4** *m/z* 424.03 $[M+H]^+$ (expected 424.11), **6** *m/z* 272.80 $[M+H]^+$ (expected 273.08) and **5-1-1** *m/z* 407.98 $[M+H]^+$ (expected 408.11)

A

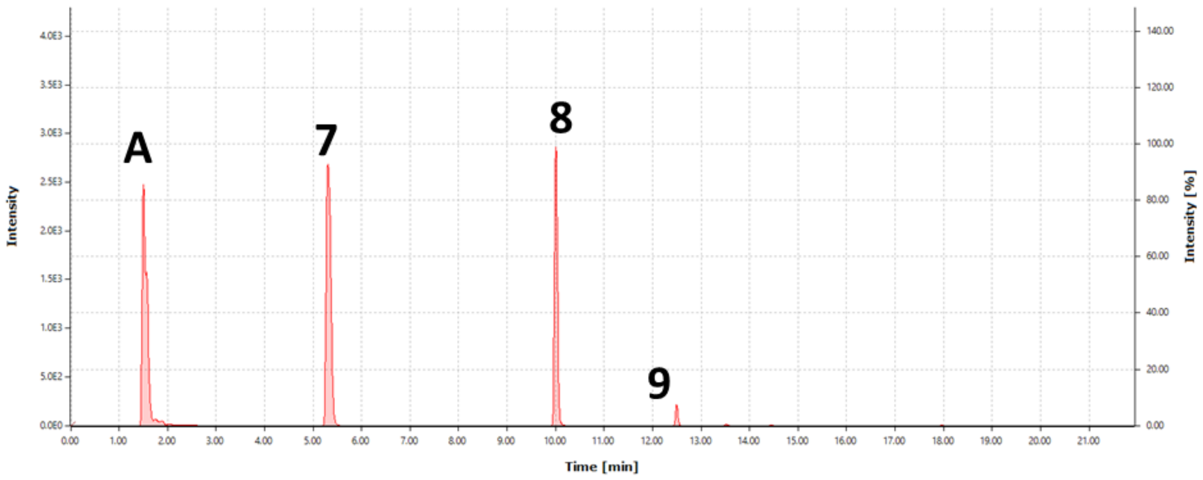

B

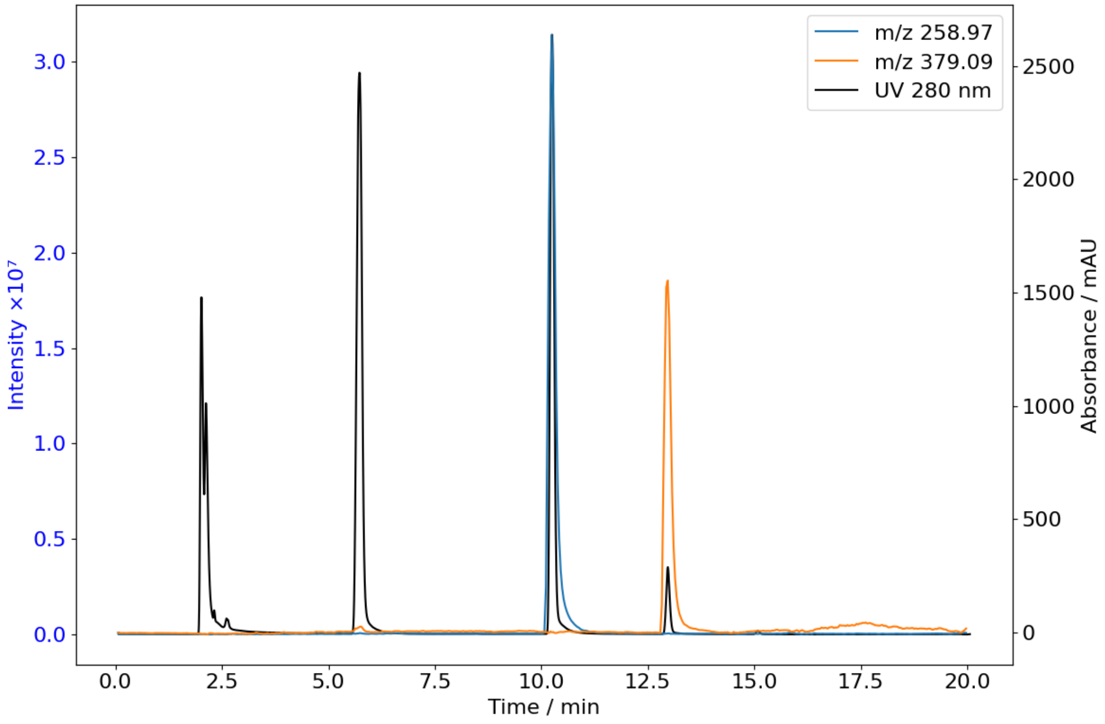

Figure S5. A: HPLC trace for ClxA-catalyzed homo-coupling of *para*-hydroxybenzoic acid **7** (**A** = ATP/AMP); **B**: Extracted Ion Chromatograms of products **8** *m/z* 258.97 [M+H]$^+$ (expected 259.06) and **9** *m/z* 379.09 [M+H]$^+$ (expected 379.07).

A

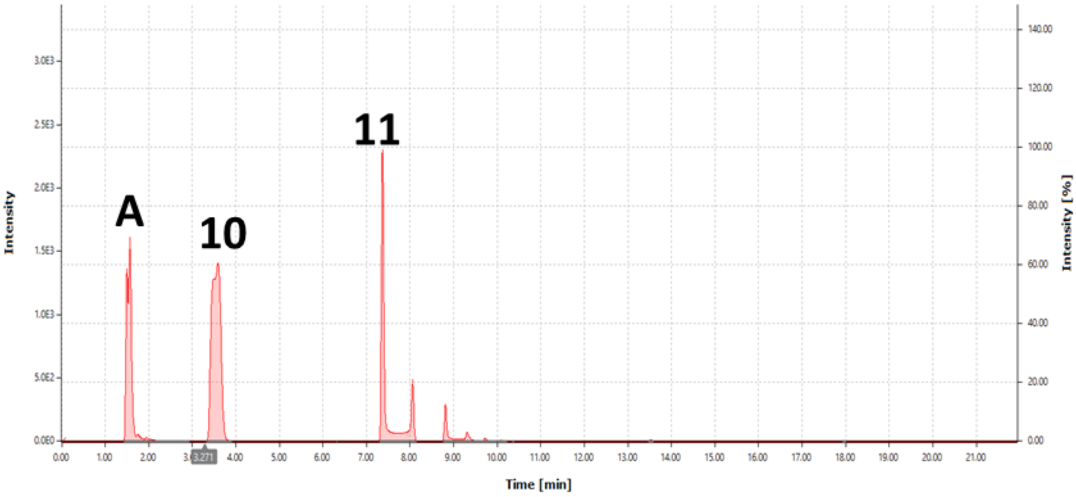

B

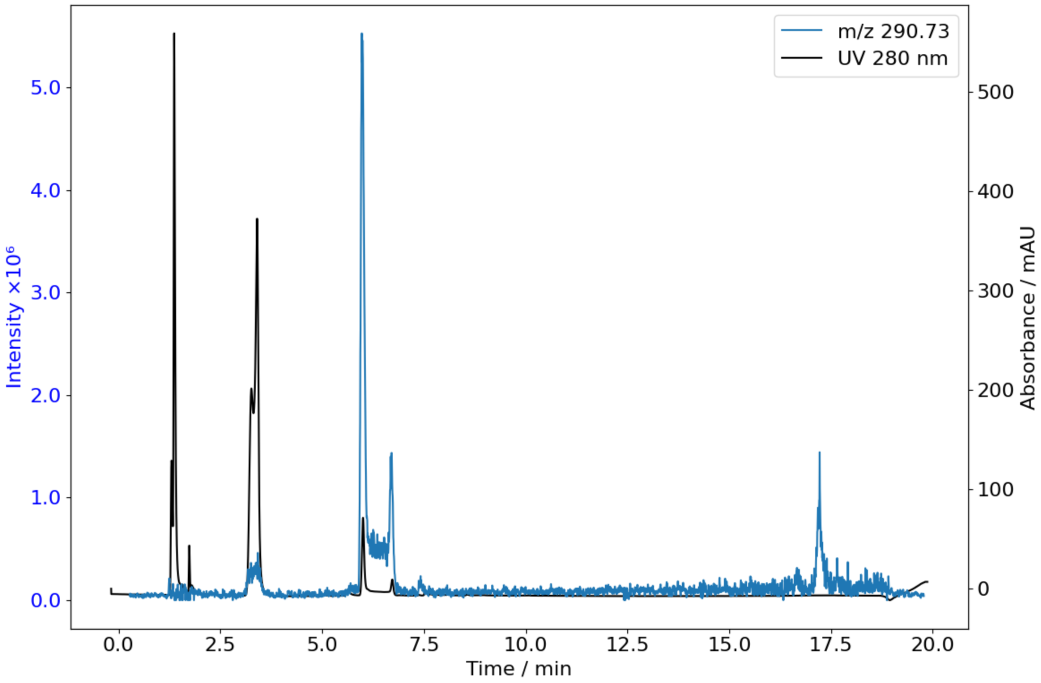

Figure S6: **A:** HPLC trace for ClxA catalyzed homo-coupling of 3,4-dihydroxybenzoic acid **10** (**A** = ATP/AMP); **B**: Extracted Ion Chromatograms of product **11** *m/z* 290.73 $[M+H]^+$ (expected 291.04).

6. X-ray Crystallography

Protein crystallisation

Initial screening of crystallization conditions was performed using commercially available INDEX (Hampton Research), PACT premier and CSSI/II (Molecular Dimensions) screens in 96-well sitting drop trays which were stored at 4 °C.

Apo- crystals of ClxA were obtained in drops containing 0.2 M $MgCl_2.6H_2O$, 0.1 M Tris pH 8.5 and 25 % (w/v) PEG 3350. Ligand complexes were obtained by co-crystallisation. 4 mM 3-amino-4-hydroxybenzoic acid **1**, derived from a 100 mM stock solution in DMSO, and 2 mM ATP, derived from a 100 mM stock solution in H_2O, were added to the ClxA solution and incubated on ice for 30 min before the trays were set up. Crystals were obtained in 0.1 M succinic acid pH 7.0 and 15 % (w/v) PEG 3350.

Crystals were harvested directly into liquid nitrogen with nylon CryoLoops™ (Hampton Research), using the mother liquor without any further cryoprotectant.

Data collection, structure solution and refinement

The datasets described in this report were collected at the Diamond Light Source, Didcot, Oxfordshire, U.K. on beamline I03. Data were processed and integrated using XDS[2] and scaled using SCALA[3] included in the Xia2[4] processing system. Data collection statistics are provided in **Table S3**. The crystals of ClxA were obtained in space group *P*1, with four molecules in the asymmetric unit; The structure of ClxA was solved by molecular replacement using MOLREP[5] with the AlphaFold[6] structure (AF-A0A1M6IZB6) as the model. The structures were built and refined using iterative cycles in Coot[7] and REFMAC,[8] employing local NCS restraints in the refinement cycles. The final structures of *apo*-ClxA and the ClxA ligand complex exhibited % R_{cryst}/R_{free} values of 26.7/30.1 and 21.0/26.7 respectively. Refinement statistics for the structures are presented in **Table S3.** The structures of *apo*-ClxA and the ClxA ligand complex have been deposited in the Protein Databank (PDB) with accession codes **9S3L** and **9S45** respectively.

Table S3. Data collection and refinement statistics for ClxA datasets. Numbers in brackets refer to data for highest resolution shells.

	ClxA (*apo*)<br>**Dataset #1**<br>**9S3L**	ClxA complex with AMP, 3,4-AHBA and adenylate<br>**9S45**
Beamline	I03	I03
Wavelength (Å)	0.73380	0.84550
Resolution (Å)	40.68-2.57 (2.64-2.57)	57.26-2.15 (2.19-2.15)
Space Group	*P*1	*P*1
Unit cell (Å)	a = 71.94; b = 79.97; c = 81.77<br>α = 90.26°; β = 95.58° γ = 103.50°	a = 73.21; b = 80.24; c = 82.74<br>α = 89.97°; β = 84.29° γ = 76.16°
No. of molecules in the asymmetric unit	4	4
Unique reflections	55692 (4566)	97660 (4799)
Completeness (%)	99.2 (98.9)	98.7 (98.9)
R_{merge} (%)	0.29 (1.18)	0.10 (0.61)
$R_{p.i.m.}$	0.17 (0.76)	0.10 (0.61)
Multiplicity	3.8 (3.4)	3.6 (3.7)
$<I/\sigma(I)>$	2.8 (0.4)	12.6 (1.9)
Overall *B* from Wilson plot ($Å_2$)	28	24
$CC_{1/2}$	0.96 (0.59)	0.99 (0.68)
R_{cryst}/ R_{free} (%)	26.7/30.1	21.0 (26.7)
r.m.s.d 1-2 bonds (Å)	0.007	0.045
r.m.s.d 1-3 angles (°)	1.523	1.892
Avge main chain B ($Å^2$)	44	29
Avge side chain B ($Å^2$)	44	32
Avge waters B ($Å^2$)	14	32
Avge Ligand B ($Å^2$)	-	44

7. Ligplot Diagram for Binding Site of the 3,4-AHBA adenylate

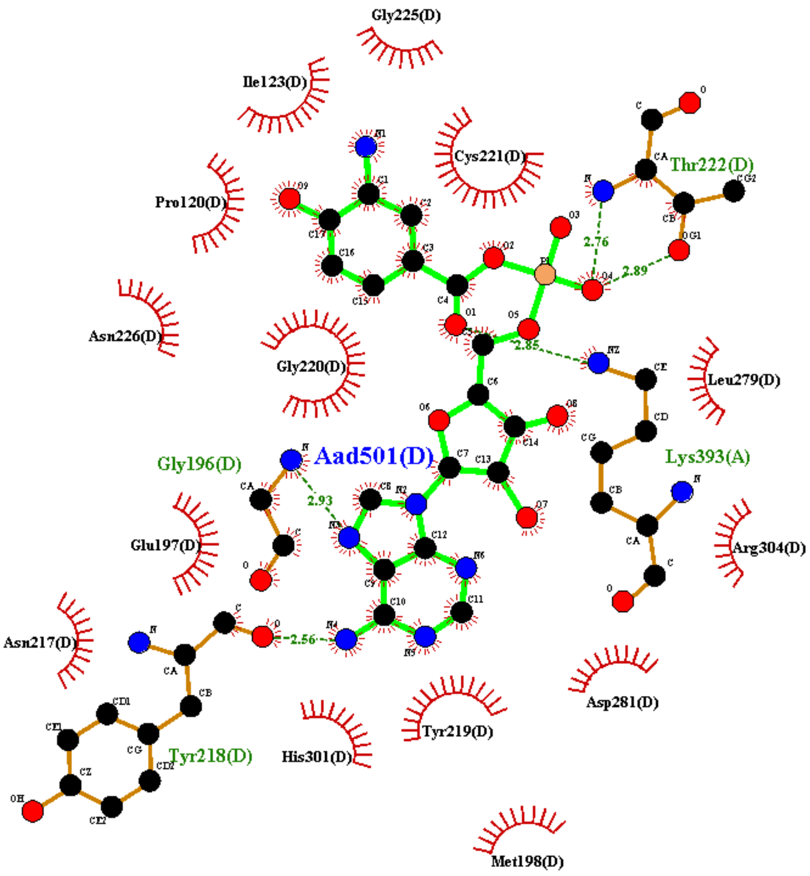

Figure S7. Ligplot diagram showing interactions of the 3,4-AHBA adenylate (Aad501) with the active site of ClxA subunit 'D'.

8. Sequence Alignments

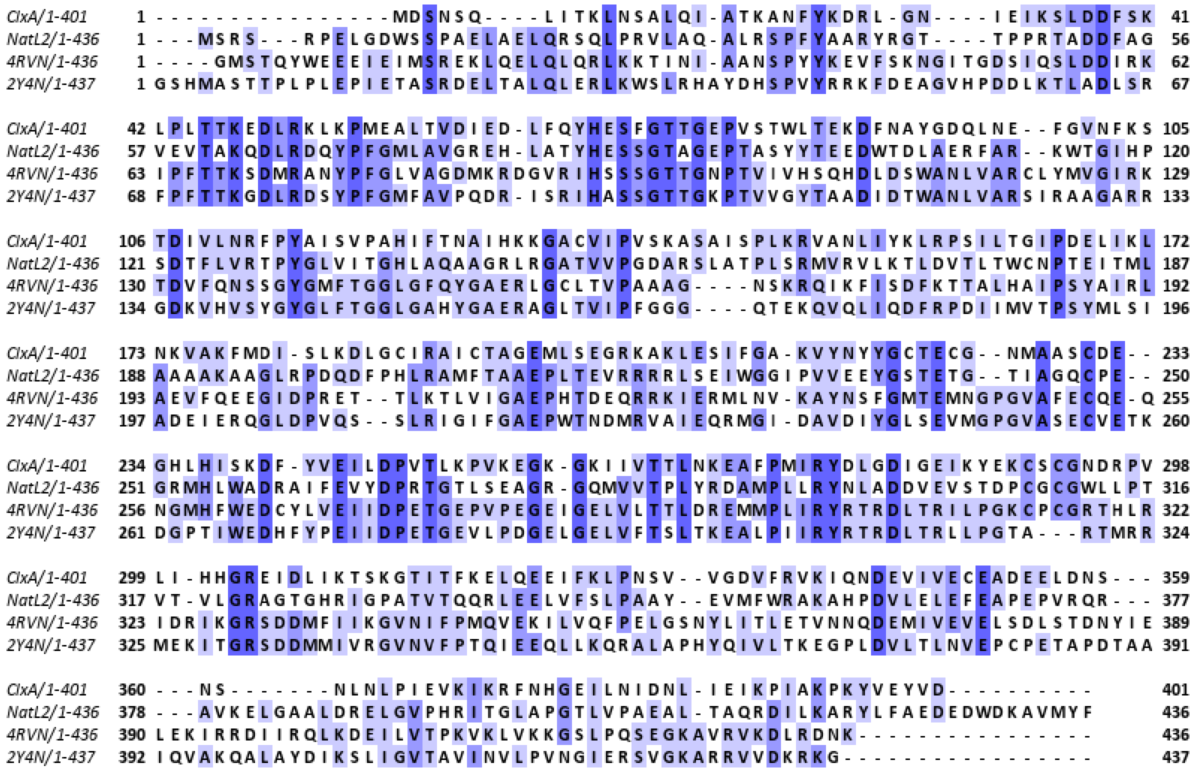

Figure S8. Sequence Alignment between ClxA, NatL2,[9] 4RVN and 2Y4N.[10]Aligment was generated using Clustal Omega.[11]

9. AutoDock

Automated docking was performed using AUTODOCK VINA 1.1.2.[12] Coordinates for the ligand were prepared using the ligand builder in COOT[7] with the relevant topology files generated in ACEDRG.[13] The appropriate pdbqt files for the dimeric model of ClxA in complex with AMP and 3-amino-4-hydroxybenzoic acid **1** located in the acyl donor pocket, and for the ligand were prepared in AUTODOCK Tools. The active site of ClxA was contained in a grid size of 50 Å × 50 Å × 50 Å (corresponding to x, y, z) with 1 Å spacing, centred around the catalytic centre at positions 44.97 Å × 1.23. Å × 47.07 Å (corresponding to x, y, z), which was generated using AutoGrid in the AUTODOCK Tools interface. The dockings were performed by VINA, therefore the posed dockings were below 2 Å rmsd. The results generated by VINA were visualised in AUTODOCK Tools 1.5.6 where the ligand conformations were assessed based upon lowest VINA energy. The top ligand conformation had a binding energy of -6.6 kcal/mol.

10. Ligplot Diagram for Modelled Binding Site for 3,4-AHBA 1 (Unl502) as an acceptor molecule

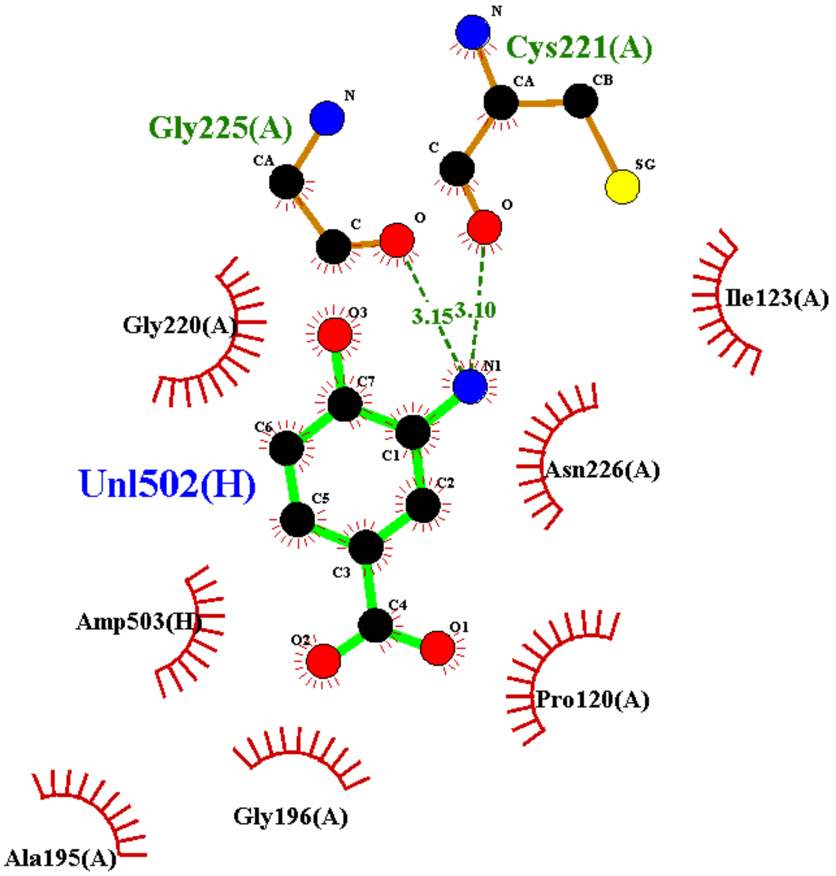

Figure S9. Ligplot diagram showing interactions of the 3,4-AHBA **1** (Unl502) with the active site of ClxA.

11. Site-Directed Mutagenesis

N226L and N226A mutations were made using the QuikChange method (Agilent). Primers are listed in **Table S4**. The K140A mutation was introduced by linearizing the plasmid, with the mutation contained on a 15 bp complementary overhang which was re-circularised using In-Fusion Snap-Assembly Master Mix (Takara Bio). The products were then digested using DpnI (New England Biolabs) according to the manufacturer's instructions.

Stellar competent cells (Takara Bio) were transformed with the resulting plasmids and grown on LB-Agar plates supplemented with 35 µg mL^{-1} kanamycin. Plasmids were extracted and purified using a Wizard Plus Plasmid Purification kit (Promega) according to the manufacturer's protocol.

Mutations were confirmed by Sanger sequencing using a standard T7 or T7-term primers (Eurofins/Genewiz).

Primer Name	Primer sequence 5' -> 3'
ClxA N226A F	GCT GTA CCG AAT GCG GTG CCA TGG CAG CAA GCT GTG
ClxA N226A R	CAC AGC TTG CTG CCA TGG CAC CGC ATT CGG TAC AGC
ClxA N226L F	CGG CTG TAC CGA ATG CGG TCT AAT GGC AGC AAG CTG TGA TG
ClxA N226L R	CAT CAC AGC TTG CTG CCA TTA GAC CGC ATT CGG TAC AGC CG
ClxA K140A F IF	GTG TCA GCA GCT TCT GCC ATC AGC CCG CTG
ClxA K140A R IF	AGA AGC TGC TGA CAC TGG AAT TAC GCA GGC ACC

Table S4. Primers Used for Site-Directed Mutagenesis experiments.

12. HPLC/MS data for biotransformations by ClxA N226L and K140

A

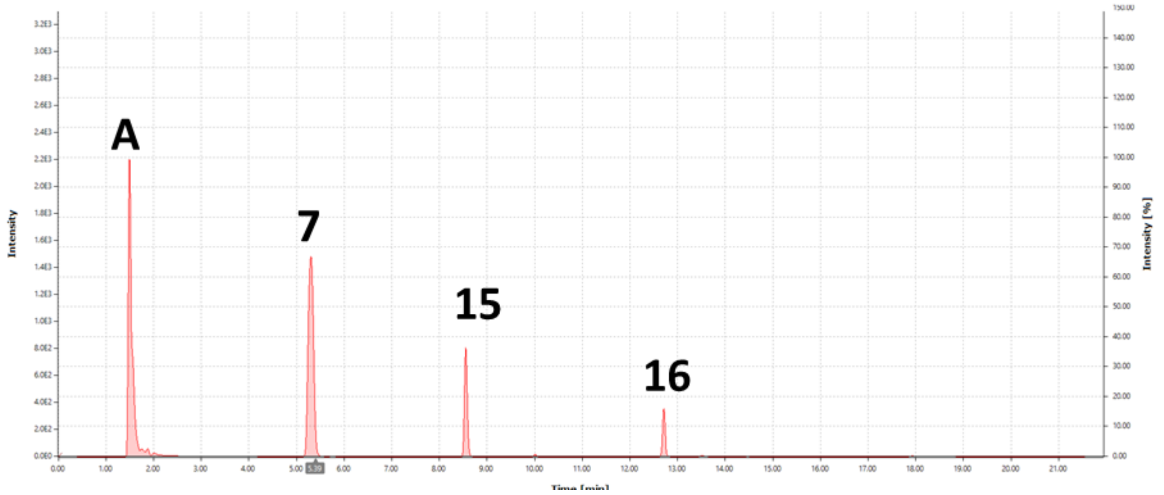

B

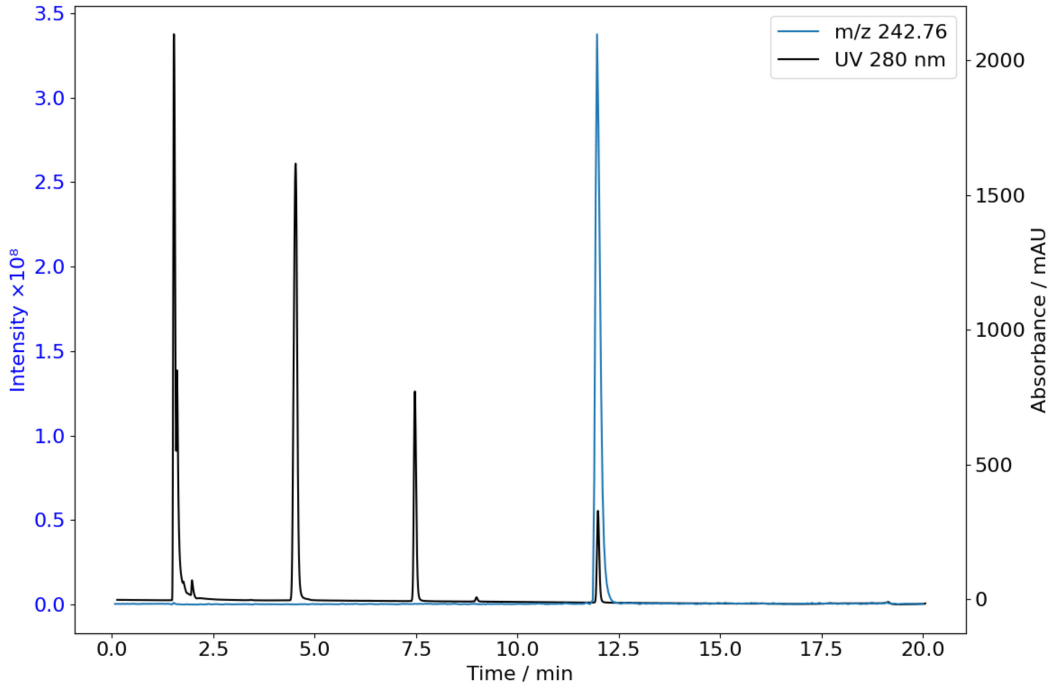

Figure S10. **A**: HPLC trace for ClxA-N226L catalysed coupling of benzoic acid **15** and 4-hydroxybenzoic acid **7** (**A** = ATP/AMP); **B.** Extracted Ion Chromatograms of product **16** *m/z* 242.76 $[M+H]^+$ (expected 243.06).

A

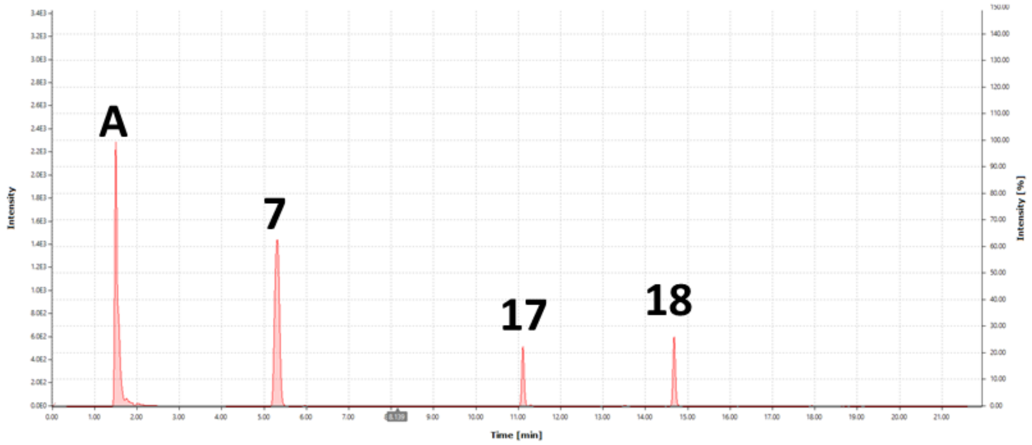

B

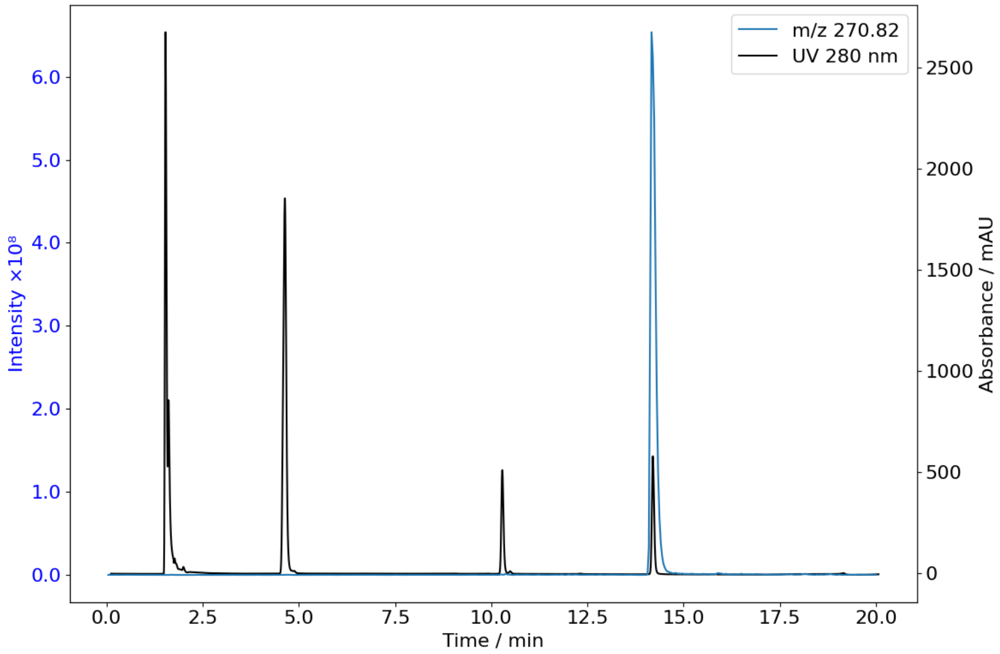

Figure S11: HPLC trace for ClxA-N226L catalysed coupling of 3,4-dimethylbenzoic acid **17** and 4-hydroxybenzoic acid **7** (**A** = ATP/AMP); **B:** Extracted Ion Chromatograms of product **18** *m/z* 270.82 $[M+H]^+$ (expected 271.09).

A

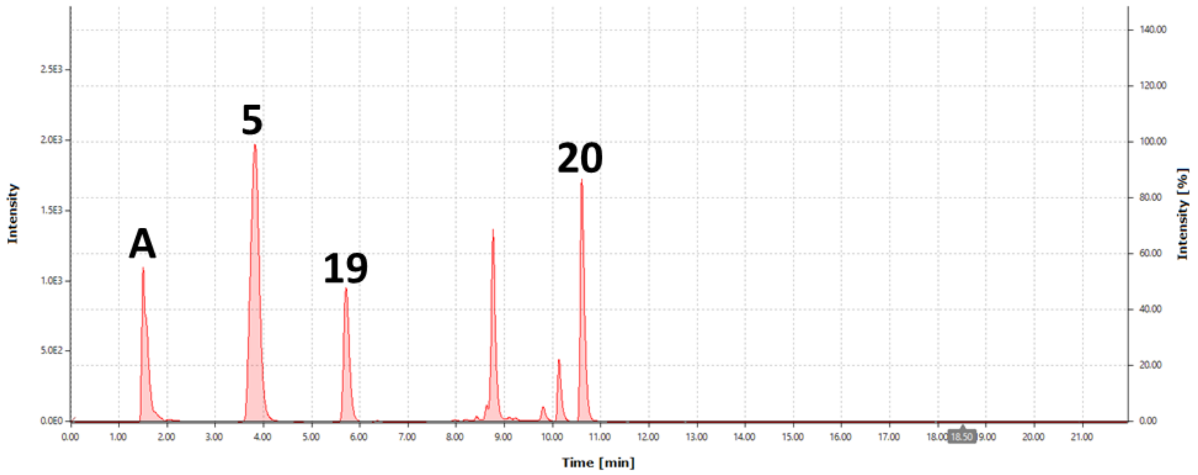

B

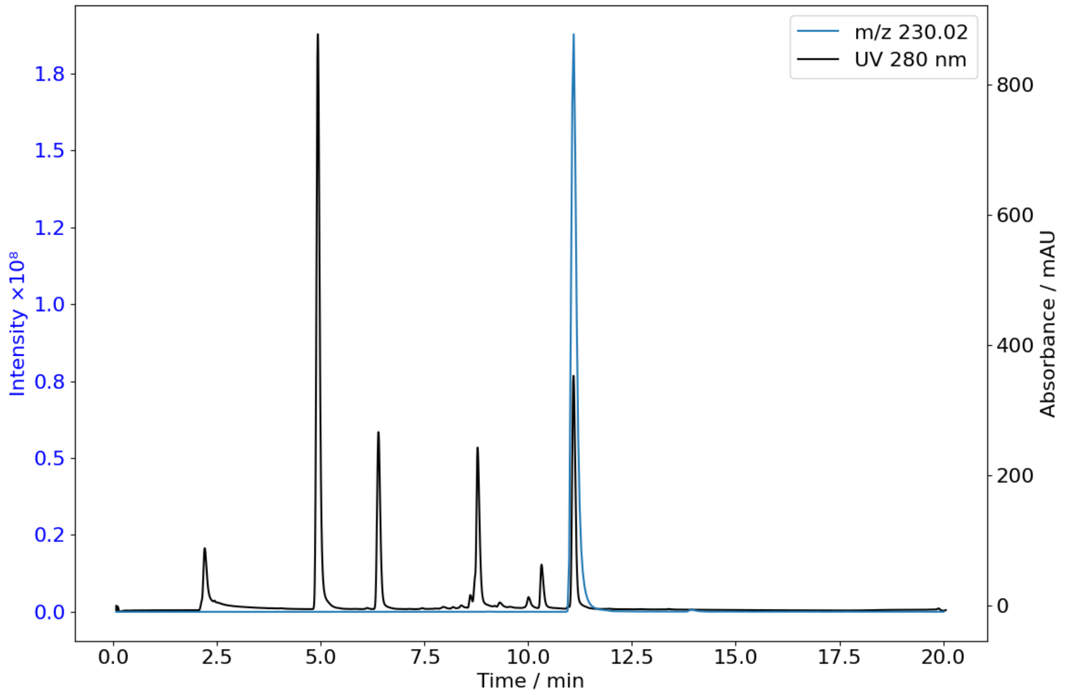

Figure S12. **A:** HPLC trace for ClxA-K140A catalysed coupling of 4-aminobenzoic acid **5** and catechol **19** (**A** = ATP/AMP); **B:** Extracted Ion Chromatograms of product **20** *m/z* 230.02 $[M+H]^+$ (expected 230.07).

A

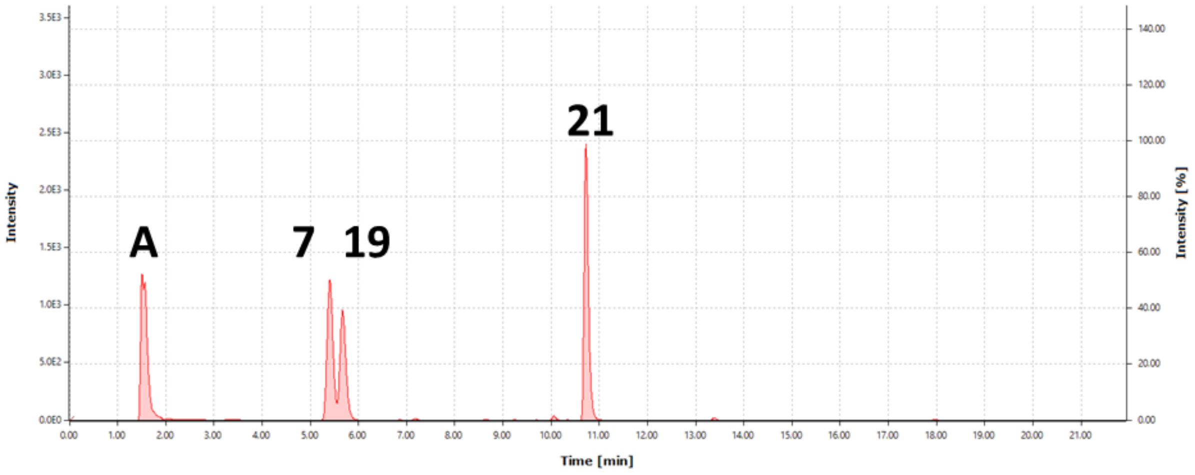

B

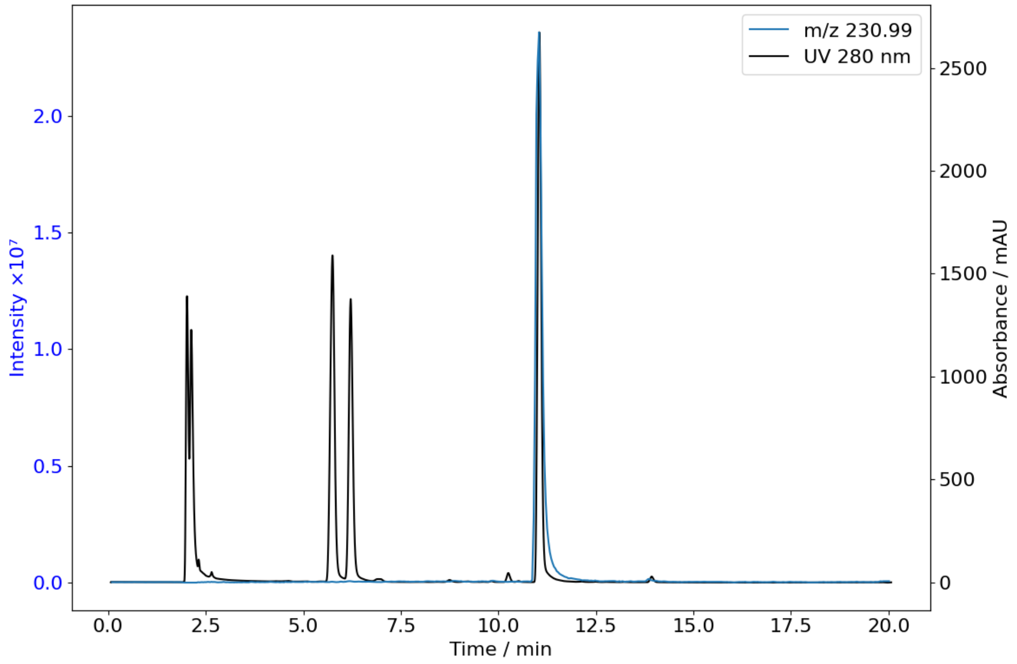

Figure S13. **A**: HPLC trace for ClxA-K140A catalysed coupling of 4-hydroxybenzoic acid **7** and catechol **19** (**A** = ATP/AMP); **B**: Extracted Ion Chromatograms of product **21** *m/z* 230.99 $[M+H]^+$ (expected 231.06).

A

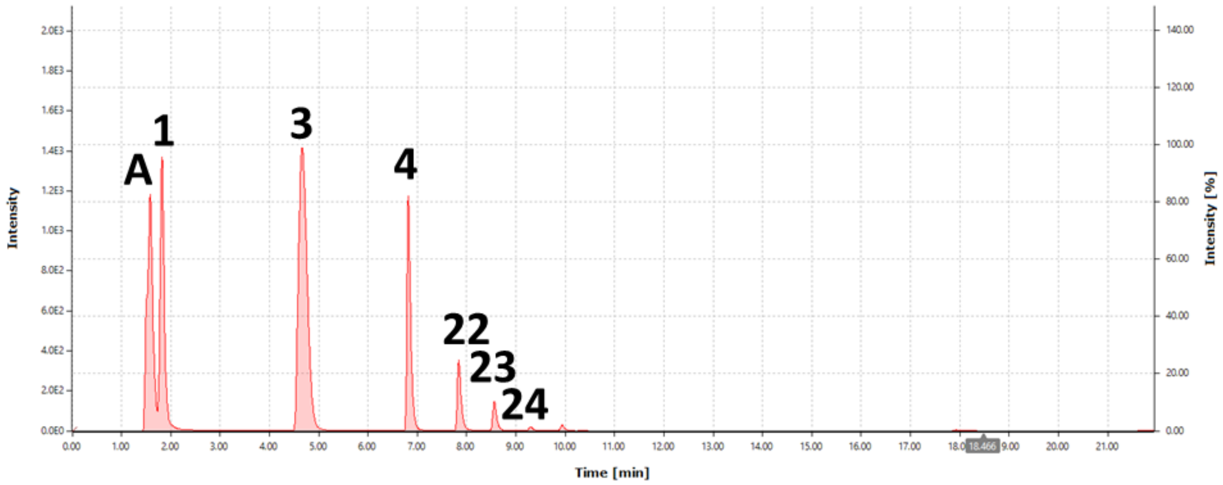

B

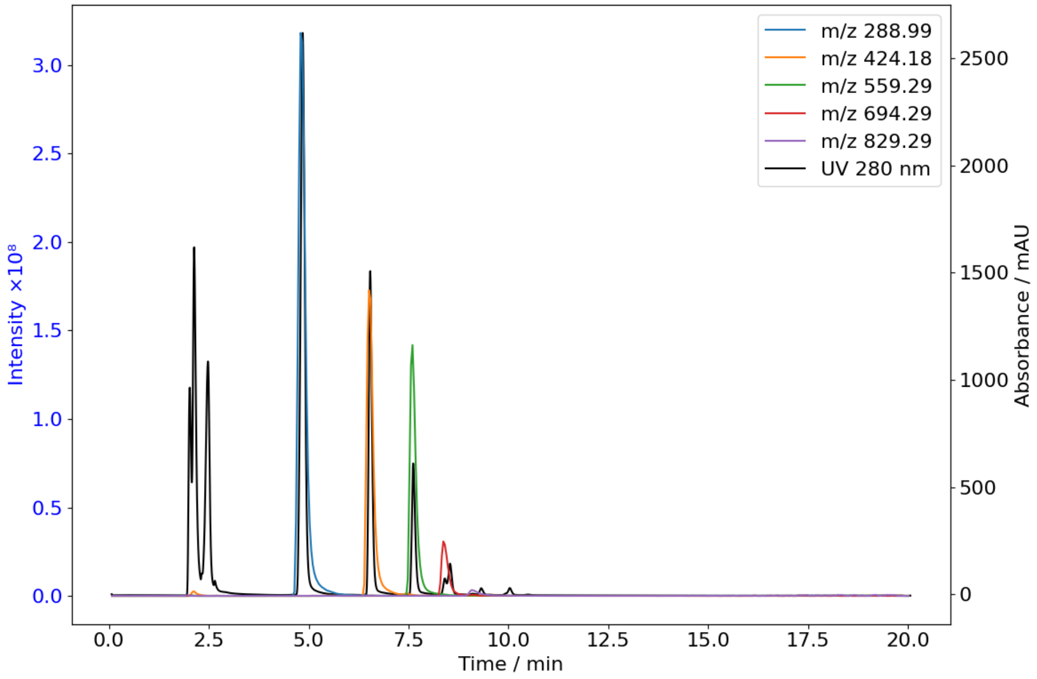

Figure S14. A: HPLC trace for ClxA-K140A catalysed coupling of 3-amino-4-hydroxybenzoic acid **1** and catechol (**A** = ATP/AMP); **B:** Extracted Ion Chromatograms of products **3**, **4**, **22** *m/z* 559 $[M+H]^+$ (expected 559.14), **23** *m/z* 694.29 $[M+H]^+$ (expected 694.17) and **24** *m/z* 829.29 $[M+H]^+$ (expected 829.20).

13. References

1. K. E. Atkin, R. Reiss, N. J. Turner, A. M. Brzozowski and G. Grogan, *Acta Crystallogr. Sect. F Struct. Biol. Cryst. Commun.*, 2008, **64**, 182-185.

2. W. Kabsch, *Acta Crystallogr. Sect. D Biol. Crystallogr.*, 2010, **66**, 125-132.

3. P. Evans, *Acta Crystallogr. Sect. D*, *Biol. Crystallogr.*, 2006, **62**, 72-82.

4. G. Winter, *J. Appl. Crystallogr.*, 2010, **43**, 186-190.

5. A. Vagin and A. Teplyakov, *J. Appl. Crystallogr.*, 1997, **30**, 1022-1025.

6. J. Jumper, R. Evans, A. Pritzel, T. Green, M. Figurnov, O. Ronneberger, K. Tunyasuvunakool, R. Bates, A. Žídek, A. Potapenko, A. Bridgland, C. Meyer, S. A. A. Kohl, A. J. Ballard, A. Cowie, B. Romera-Paredes, S. Nikolov, R. Jain, J. Adler, T. Back, S. Petersen, D. Reiman, E. Clancy, M. Zielinski, M. Steinegger, M. Pacholska, T. Berghammer, S. Bodenstein, D. Silver, O. Vinyals, A. W. Senior, K. Kavukcuoglu, P. Kohli and D. Hassabis, *Nature*, 2021, **596**, 583-589.

7. P. Emsley and K. Cowtan, *Acta Crystallogr. Sect. D Biol. Crystallogr.*, 2004, **60**, 2126-2132.

8. G. N. Murshudov, A. A. Vagin and E. J. Dodson, *Acta Crystallogr. Sect. D Biol. Crystallogr.*, 1997, **53**, 240-255.

9. H. Song, C. Rao, Z. Deng, Y. Yu, J. H. Naismith, *Angew. Chem. Int. Ed.*, 2020, **59**, 6054-6061.

10. A. Law, M. J. Boulanger, *J. Biol. Chem.* 2011, **286**, 15577-15585.

11. F. Sievers A. Wilm, D.G. Dineen, T.J. Gibson, K. Karplus, W. Li, R. Lopez, H. McWilliam, M. Remmert, J. Söding, J.D. Thompson and D. Higgins, *Mol. Syst. Biol.* 2011, **7**, 539.

12. O. Trott and A.J. Olson, *J. Comput. Chem.* 2009, **31**, 455-461.

13. F. Long, R. A. Nicholls, P. Emsley, S. Graǽulis, A. Merkys, A. Vaitkus, G. N. Murshudov, *Acta Crystallogr. D Struct. Biol.* 2017, **73**, 112-122.
